# Efficient and Green Isolation of Keratin from Poultry Feathers by Subcritical Water

**DOI:** 10.3390/polym15122658

**Published:** 2023-06-12

**Authors:** Mojca Škerget, Maja Čolnik, Lidija Fras Zemljič, Lidija Gradišnik, Tanja Živković Semren, Blanka Tariba Lovaković, Uroš Maver

**Affiliations:** 1 Laboratory for Separation Processes and Product Design, Faculty of Chemistry and Chemical Engineering, University of Maribor, Smetanova 17, 2000 Maribor, Slovenia; maja.colnik@um.si; 2 Laboratory for Characterization and Processing of Polymers, Faculty of Mechanical Engineering, University of Maribor, Smetanova 17, 2000 Maribor, Slovenia; lidija.fras@um.si; 3 Institute of Biomedical Sciences, Faculty of Medicine, University of Maribor, Taborska 8, 2000 Maribor, Slovenia; lidija.gradisnik@um.si (L.G.); uros.maver@um.si (U.M.); 4 Analytical Toxicology and Mineral Metabolism Unit, Institute for Medical Research and Occupational Health, Ksaverska Cesta 2, 10000 Zagreb, Croatia; tzivkovic@imi.hr (T.Ž.S.); btariba@imi.hr (B.T.L.); 5 Department of Pharmacology, Faculty of Medicine, University of Maribor, Taborska 8, 2000 Maribor, Slovenia

**Keywords:** poultry feathers, subcritical water hydrolysis, keratin, physico-chemical characterization, cytotoxicity

## Abstract

The isolation of keratin from poultry feathers using subcritical water was studied in a batch reactor at temperatures (120–250 °C) and reaction times (5–75 min). The hydrolyzed product was characterized by FTIR and elemental analysis, while the molecular weight of the isolated product was determined by SDS-PAGE electrophoresis. To determine whether disulfide bond cleavage was followed by depolymerization of protein molecules to amino acids, the concentration of 27 amino acids in the hydrolysate was analyzed by GC/MS. The optimal operating parameters for obtaining a high molecular weight protein hydrolysate from poultry feathers were 180 °C and 60 min. The molecular weight of the protein hydrolysate obtained under optimal conditions ranged from 4.5 to 12 kDa, and the content of amino acids in the dried product was low (2.53% *w*/*w*). Elemental and FTIR analyses of unprocessed feathers and dried hydrolysate obtained under optimal conditions showed no significant differences in protein content and structure. Obtained hydrolysate is a colloidal solution with a tendency for particle agglomeration. Finally, a positive influence on skin fibroblast viability was observed for the hydrolysate obtained under optimal processing conditions for concentrations below 6.25 mg/mL, which makes the product interesting for various biomedical applications.

## 1. Introduction

Recycling keratin-rich waste biomasses such as hair, wool, feathers, horns, hooves and hides have been the objective of numerous research studies and several review articles have been published in the last decade discussing keratin structure [1,2,3,4,5,6,7] the physicochemical and biological properties of keratin [1,2,3,4,6,7,8,9] and its potential applications [1,2,3,4,6,7,8,9,10,11,12] as well as various methods for isolating keratin from different biomasses. These methods include chemical hydrolysis (i.e., alkaline and acid hydrolysis, reduction, sulfitolysis, and oxidation) [1,2,3,4,6,7,8,12,13], enzymatic and microbial treatment [1,2,4,5,6,10,11], dissolution in ionic liquids [2,3,4,8] or deep eutectic solvents [3], microwave irradiation [2,4,6], hydrothermal processing such as steam explosion, and use of superheated water [1,2,3,4,6,10,11]. Due to the specific properties of keratin such as biodegradability, mechanical strength, good cell adhesion, and biocompatibility, as well as good processing characteristics that allow the production of various forms such as gels, films, beads, fibers, nano/microparticles, and keratin could find numerous applications in food, cosmetic, pharmaceutical, medical, textile, composite, agricultural, and other industries. A green approach that has recently been considered very promising for recycling proteinaceous waste is thermal hydrolysis using water, i.e., hydrothermal processing. Subcritical water (SubCW), i.e., water at temperatures above 100 °C and elevated pressure, can serve as a solvent, reactant, and catalyst at the same time. This is possible due to its specific properties such as lower polarity and higher ionization constant [14,15,16,17]. In the reactions with biomass, it causes the breaking of chemical bonds. The advantage of this method is that the process is environmentally friendly. Namely, SubCW hydrolysis operates without harmful chemicals and expensive enzymes or bacteria [18]. Moreover, by using thermal hydrolysis with SubCW under certain conditions, it is possible to destroy pathogens, making this method suitable for processing potentially infectious tissues [18]. Considering all mentioned, using SubCW to isolate keratins (proteins) seems highly challenging. It requires in-depth study and optimization of processing conditions to minimize protein depolymerization and further degradation of monomers and to maximize the yield of high molecular weight proteins. In addition, keratins from different sources are structurally different, suggesting that different optimal conditions for hydrothermal processing must be used in the case of different keratin sources due to the different amounts of covalent intermolecular disulfide bonds.

Chicken feathers contain about 91% protein (keratin), 1% lipids, and 8% water [9]. The main amino acids in feathers keratin are serine, glutamic acid, glycine, leucine, proline, alanine, and valine [8]. The sulfur-containing cysteine/cystine content is 7%. The molecular weight of feather keratin is about 10,500 Da [7]. Cysteine residues are oxidized to form inter- and intramolecular disulfide bonds, stabilizing and cross-linking the filaments to provide high stability to the protein [8].

To date, several studies on the hydrolysis of keratin-rich biomass using SubCW have been performed. These studies mainly aimed at obtaining protein hydrolysates rich in small molecules (peptides and amino acids) for potential applications as fertilizers, animal feeds, biofuels, or as building blocks for the synthesis of novel polymers [14,16,17,19,20,21,22,23]. On the other hand, studies on the recovery of protein hydrolysates with high molecular weight are rare. In a recent work by Di Domenico Ziero et al. [20], SubCW hydrolysis of poultry feathers to recover amino acids was evaluated by performing experiments in a semi-continuous flow-through reactor at temperatures ranging from 210 to 250 °C and a pressure of 150 bar. It was found that the conversion of proteins to amino acids required higher temperatures (250 °C) and low flow rates to ensure sufficient residence time. In addition, the yields obtained using water hydrolysis were higher than using conventional methods (alkaline hydrolysis or enzymatic method). Tasaki [24] developed a two-step hydrolysis process to extract keratin from hog hair. The first step, which was carried out at 140 °C, was used to swell and denature the keratin protein network in the intermediate filaments, while the second step, in which the temperature was varied from 100 to 220 °C with a reaction time of 1 h, and was used to cleave the disulfide bonds that bind the keratin fibrils together. The two-step procedure yielded nearly 70% of the keratin with a wide range of MW distribution between 20 kDa and 100 kDa. In the work of Bhavsar et al. [19], superheated water hydrolysis of wool at temperatures from 140 to 170 °C was compared to conventional alkaline hydrolysis using KOH and CaO, and it was found that both hydrolysates contained low molecular weight proteins and amino acids. In our previous work [25], SubCW extraction of wool to obtain keratin was studied at temperatures from 150 °C to 250 °C and reaction times between 5 min and 75 min. In this study, much higher yield of keratin was obtained than with other chemical methods. The maximum yield (90.3%) was obtained at 180 °C and 60 min and the molecular weight of the extracted proteins ranged from 4 kDa to 14 kDa. Hydrolysis of feathers in water under specific pressure–temperature conditions (180–220 °C and pressure about 22 bar), where the water is in the vapor state, was studied by Yin et al. [23], and peptides with molecular weights of 1.0 and 1.8 kDa were obtained. It was observed that these oligopeptides self-assemble into a hierarchical architecture under suitable conditions [23].

To our knowledge, no studies on the recovery of keratin from poultry feathers using subcritical water in a liquid state have been published to date. Since higher molecular weight polymers are preferred for further processing by electrospinning or 3D printing to produce biomaterials for biomedical applications, this study aimed to evaluate the potential of SubCW hydrolysis to recover high molecular weight keratin from poultry feathers in high yield. The influence of operating conditions such as temperature and reaction time on the yield and molecular weight of the obtained products was studied. The obtained solution was analyzed for amino acid content to determine whether and to what extent depolymerization had occurred. The colloidal nature of the obtained hydrolysate was studied using zeta potential and particle size measurements. Finally, considering the potential use of keratin in biomedical applications, its influence on skin fibroblast viability was also analyzed.

## 2. Materials and Methods

### 2.1. Materials

Poultry feathers were obtained from Perutnina Ptuj d.d. (Ptuj, Slovenia). Before use, the industrially pre-cleaned waste feathers were placed in a mesh bag and washed in a washing machine at 60 °C with non-ionic detergent (Sandoclean PC, Sandoz, Basel, Switzerland). The optimized cleaning procedure was established earlier and is described in detail in the paper of Strnad et al. [26]. Until use, the feathers were kept at room temperature. Coomassie brilliant blue G-250 and acetic acid were supplied by Merck, Germany. Lonza™ ProSieve™ protein marker was supplied by Fisher Scientific (Waltham, MA, USA). 3X blue stained deposition buffer for SDS-PAGE electrophoresis and dithiothreitol (DTT) were supplied by BioLabs Inc (Ipswich, MA, USA. Trizma^®^ base, sodium dodecyl sulfate, ammonium persulfate, glycine, methanol, 2-propanol, and hydrochloric acid were supplied by Sigma-Aldrich (Steinheim, Germany).

### 2.2. Subcritical Water Extraction

A 75 mL high-temperature, high-pressure batch reactor (series 4740 Stainless Steel, Parr Instruments, Moline, IL, USA) was used to isolate keratin from feather waste with SubCW [25]. Experiments were performed at different temperatures ranging from 150 to 250 °C and at different reaction times from 15 to 75 min. For each experiment, the feathers (2 g) and water (40 mL) were filled into the reactor (the ratio of material to water was 1/20 g/mL). The reactor was then purged three times with an inert gas (N_2_) to prevent possible oxidation of the products. The initial N_2_ pressure in the reactor was set at 20 bar. The temperature was regulated with a heating wire, and a 600 rpm magnetic stirrer was used to mix the reaction mixture. After reaction, the autoclave was rapidly cooled. The post-reaction mixture was filtered by vacuum filtration, and the aqueous solution was collected. A total of 10 mL of the liquid product was evaporated using a rotary evaporator, and the extraction yield (*η*) was calculated according to Equation (1).

(1) $$\eta \left(\%\right)=\frac{m_{\mathrm{dry product}}\left(g\right)}{m_{\mathrm{feathers}}\left(g\right)}\cdot 100\%$$

The remaining aqueous solution was stored in the freezer for further analysis. The experiments were conducted two times, and the results are presented as mean values with the standard deviation.

### 2.3. SDS-PAGE Electrophoresis

SDS-PAGE gel electrophoresis was used to determine the molecular weight of the extracted products. In order to have a good resolution of keratin fractions by molecular weight, 15% acrylamide/bisacrylamide (Acryl/Bis) separation gel and 6% Acryl/Bis stacking gel were prepared. A total of 20 µL of protein sample (0.4–0.6 mg/mL) was added to 20 µL of loading buffer, which consisted of a mixture of Tris-HCL, SDS, glycerol, bromophenol blue, and DTT. Then, the sample-loading buffer mixture was incubated at 95 °C for 3–5 min. Afterwards, 10 µL of sample and 8 µL protein marker (mixture of 12 recombinant, highly purified proteins with molecular weights from 4.6 to 300 kDa) were pipetted into the bottom of the gel wells. Electrophoresis was performed at 150 V for 90 min with Tris-glycine-SDS running buffer. After completion of electrophoresis, the gels were washed with Milli-Q water, stained with Coomassie blue, destained in acetic acid solution until a clear background was obtained, and dried on the filter paper using a vacuum pump.

### 2.4. Analysis of Amino Acids

Concentrations of 27 amino acids (alanine, allo-isoleucine, asparagine, aspartic acid, glutamic acid, glutamine, glycine, glycyl-proline, histidine, hydroxylysine, isoleucine, leucine, lysine, methionine, ornithine, phenylalanine, proline, sarcosine, serine, thioproline, threonine, tryptophan, tyrosine, valine, α-aminoadipic acid, α-aminobutyric acid, α-aminopimelic acid) were measured in the obtained aqueous solutions by Trace 1300 gas chromatograph (Thermo Scientific, Milan, Italy) coupled to a ITQ 700 ion trap mass spectrometer (Thermo Scientific, Austin, TX, USA). Sample pre-treatment and GC-MS analysis were carried out using EZ:faastTM amino acid analysis kit (Phenomenex, Torrance, CA, USA), according to the manufacturer’s instructions and as described in Badawy et al. [27]. The injection port was operated in split mode (1:10) at 250 °C. A ZB-AAA GC column (10 m × 0.25 mm ID, 0.25 μm film thickness) was used for chromatographic separation, with He as a carrier gas at a constant flow rate of 1.1 mL/min. The initial oven temperature was set at 100 °C and raised to 320 °C at a rate of 30 °C/min. The operating conditions for the MS system were as follows: transfer line temperature 180 °C, electron ionization energy 70 eV, ion source temperature 240 °C. Data acquisition was obtained in full scan mode with scan range of *m/z* 45–450.

### 2.5. FTIR-Analysis

An IRAffinity-1 FTIR spectrophotometer (Shimadzu, Kyoto, Japan) equipped with an attenuated total reflectance cell (ATR) was used to analyze the molecular structure of feathers (calamus and vane) and the isolated solid product. The spectra of the feathers and a dried sample of isolated keratin were recorded in the range of 4000–400 cm^−1^ at a resolution of 16 cm^−1^ for a total of 30 scans. The background of the spectrum was subtracted before each analysis. High-performance IR software was used to analyse the obtained spectra.

### 2.6. Elemental Analysis

Elemental analysis of feathers and obtained solid product was performed using a Perkin Elmer 2400 Series II System Analyzer (Waltham, MA, USA), and the content of carbon, hydrogen, nitrogen, and sulfur was determined.

### 2.7. Particle Size and Zeta Potential Measurements

The particle size and zeta potential of the obtained solution were determined as a function of pH, i.e., zeta potential measurements ranged from pH = 3 to pH = 10, while particle size was measured at pH = 3, 7, and 9. Particle size and zeta potential were measured using a Litesizer 500, type FM10, Austria and an Omega cuvette Mat. No. 225288. The instrument allows the measurement of particle sizes ranging from 0.3 nm to 10 µm. The pH of the isolated solution was automatically adjusted with NaOH and HCl.

### 2.8. Cytotoxicity Assay

Since the literature does not report any data about poultry waste keratin, the first tests were performed with a higher initial concentration of the sample, close to its solubility (50 mg/mL). For this purpose, the freeze-dried sample of isolated keratin was diluted in cell culture medium (Advanced DMEM cell culture medium supplemented with 5 wt.% Foetal Bovine Serum (FBS), ThermoFisher Scientific, Waltham, MA, USA). To evaluate the cytotoxicity of the isolated keratin, the as-prepared sample was diluted in a series of dilutions (1:2, 1:4, 1:8, 1:16) according to ISO10993-12:2012 and ISO10993-5:2009. The different dilutions were obtained by serial diluting the base sample with cell culture medium. Cell culture medium (ADMEM + 5 wt.% FBS) was used as a positive control.

Skin fibroblasts (specifically Detroit 551-CCL-110 cells obtained from ATCC in the UK) were cultured in Advanced DMEM cell culture medium, which was supplemented with 5% FBS, 100 IU/mL penicillin, 0.1 mg/mL streptomycin, and 2 mM L-glutamine from Thermo Fisher Scientific, based in MA, USA. The cells were maintained at a temperature of 37 °C and in an atmosphere containing 5% CO_2_.

To assess cytotoxicity, the MTT cell viability assay protocol was employed, which measures cell viability by evaluating metabolic activity. The skin fibroblasts were seeded in 96-well plates at a density of 10,000 cells per well, with a volume of 100 µL per well. They were then cultured for 24 h. After this initial incubation period, the cells were treated with various sample dilutions as well as a control. Following exposure for an additional 24 h, the cells were incubated with a 10% MTT solution obtained from Sigma-Aldrich in Missouri, USA. This incubation was carried out for 3 h at a temperature of 37 °C. Formazan formation, which indicates cell viability, was determined for each treatment concentration and compared to the control cultures. A decrease in the number of viable cells led to a corresponding decrease in their metabolic activity. To determine cell viability, the optical density at 570 nm was quantified using a multiplate reader called Varioskan, manufactured by Thermo Fisher Scientific. Prior to measuring the optical density, the MTT solution was removed, and the cells were resuspended in 100 µL of DMSO (also from ThermoFisher Scientific, Waltham, MA, USA). Each experiment was repeated four times. Viability above 70% in comparison to the control sample was considered non-cytotoxic. The results are presented as average values with standard errors. Statistical analysis was conducted using one-way analysis of variance (ANOVA), and significance was defined as *p* < 0.05, denoted as * using Bonferroni analysis [28].

## 3. Results and Discussion

### 3.1. Yield of Extraction

**Figure 1:**
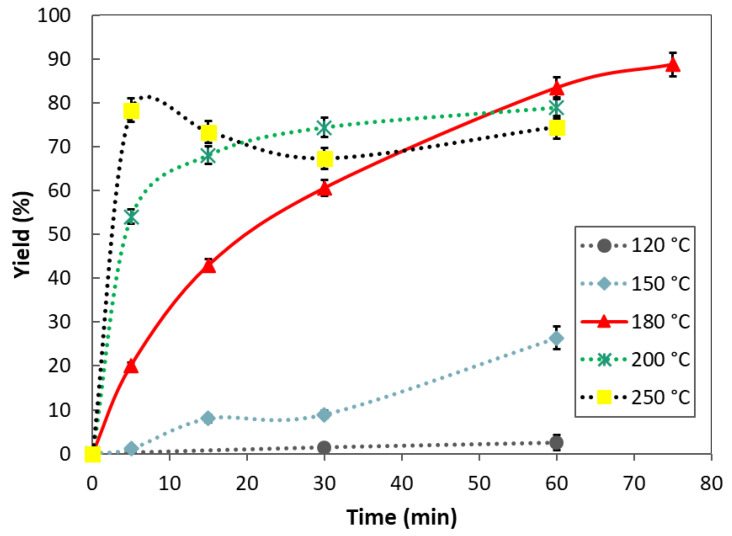
Yield of SubCW extraction of poultry feathers at different reaction conditions.

The extraction yield depends on the temperature and reaction time, as shown in Figure 1. At temperatures of 120 °C and 150 °C, the extraction rate was low, and the extraction yield reached 2.5% and 26.4% after 60 min, respectively. By increasing the temperature to 180 °C, the extraction rate increased significantly and continued to increase when the temperature was further increased to 250 °C. The maximum extraction yield (88.7%) was obtained at a temperature of 180 °C and a reaction time of 75 min, showing that the extraction efficiency of keratin was high (i.e., almost 98% considering that feathers contain 91 % keratin). At the highest temperature studied (250 °C), the yield of keratin initially increases and reaches a maximum, and then decreases, probably as a result of further decomposition of keratin to oligomers, free amino acids, and volatiles [25,29]. Similar results were obtained in our previous work for extracting keratin from waste wool using SubCW, where the extraction yield at 150 °C and 60 min was 25.6%, and the maximum yield at 180 °C and 60 min was 90.3% [25].

### 3.2. FTIR Analysis of Feathers and Hydrolysate

**Figure 2:**
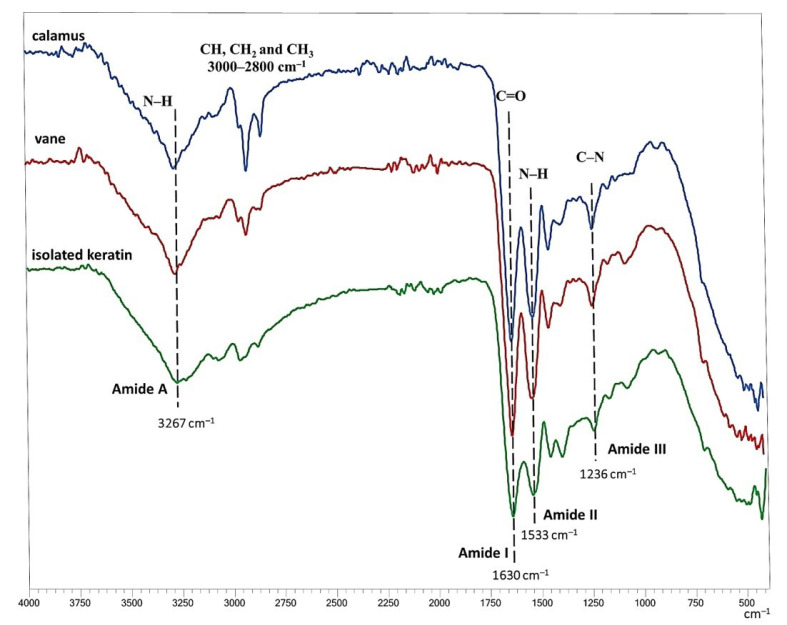
FTIR spectrum of keratin isolated from feathers with SubCW at 180 °C and a reaction time of 60 min.

FTIR analysis provides information about the chemical structure of proteins. Therefore, the FTIR spectrum of the isolated dry product (keratin) was compared with the FTIR spectra of untreated feathers (calamus and vane) (Figure 2). In general, the characteristic absorption bands of the FTIR spectra of proteins originating from specific stretching and bending vibrations of the protein backbone that are suitable for secondary structure analysis are the amide I band at 1600–1700 cm^−1^, which is mainly due to the C=O stretching vibration, the amide II band at 1480–1580 cm^−1^, which is due to N-H bending and C-H stretching vibrations, the amide III band at 1200–1300 cm^−1^, attributed to N-H and C=O bending and C-N stretching vibrations, and the amide A band at about 3300 cm^−1^, which is due to hydrogen-bonded NH stretching vibrations [30]. The frequency of this band depends on the strength of the hydrogen bonding [30]. The characteristic bands at 2850–2930 cm^−1^ are mainly due to alkyl CH stretching [31].

Figure 2 shows that the FTIR absorption spectra of the calamus and vane of untreated feathers and the spectra of the isolated keratin are similar, indicating that hydrolysis in SubCW did not alter the chemical structure and secondary structure to a detectable extent. In all cases, the characteristic bands appear at 3267 cm^−1^ for amide A, 1630 cm^−1^ for amide I, 1533 cm^−1^ for amide II, and 1236 cm^−1^ for amide III. Peaks occurring between 2800 cm^−1^ and 3000 cm^−1^ are attributed to the methylene stretching vibrations of the CH, CH_2,_ and CH_3_ functional groups [32]. Some differences, occurring probably due to the side chains in amino acids or hydrocarbon chains in lipids, are observed in the alkyl CH -stretching range at 2800 cm^−1^ to 3000 cm^−1^, where the peak intensity is lower for isolated keratin than for parts of untreated feathers (calamus and vane).

### 3.3. CHNS Elemental Analysis of Feathers and Hydrolysate

The elemental analysis results for feathers are comparable to those reported in the literature [33,34,35]. They show no significant but minor differences between the feathers’ elemental weight fractions (C/N/H) and the hydrolyzed product. Namely, feathers contain 49.26% C, 8.96% H, 15.49% N, and 3.19% S. In comparison, the dried product obtained from feathers with SubCW at 180 °C and 60 min contained 45.66% C, 7.35% H, 15.01% N, and 1.61% S.

Tuna et al. [35] reported that the carbon-to-nitrogen (C/N) ratio indicates the degree of degradation due to chemical changes during the thermal process. A comparison of C/N ratio before (3.18) and after (3.04) hydrothermal treatment indicates that keratin in feathers was not subjected to major degradation as the two values are not considerably different. Based on the nitrogen content, the protein content can be quantified by multiplying the nitrogen content by a factor which corresponds to the average nitrogen content. According to the official AOAC methods, the conversion factor is 6.25 [20,36]. Thus, the elemental analysis results show that the difference in protein content between the hydrolyzed product and the untreated feathers is not very large, only 3%.

In addition, elemental analysis is useful to characterize the content of S present in feather keratin in cystine disulfide bonds (S-S), cysteine-free thiol groups (SH), or methionine. Since the methionine content is low and it is assumed that the amount of free thiol groups is less than 5%, almost all of the sulfur is considered to locate in disulfide bonds [37]. Elemental analysis showed that the S content in the product obtained after hydrolysis in SubCW is lower than in the untreated feathers, which is probably due to cleavage of the disulfide bonds. As described in the literature, the free thiol groups then probably react further, for example, to form sulfoxyl compounds or sulfur-containing volatiles [38].

### 3.4. Molecular Weight of Hydrolysate

**Figure 3:**
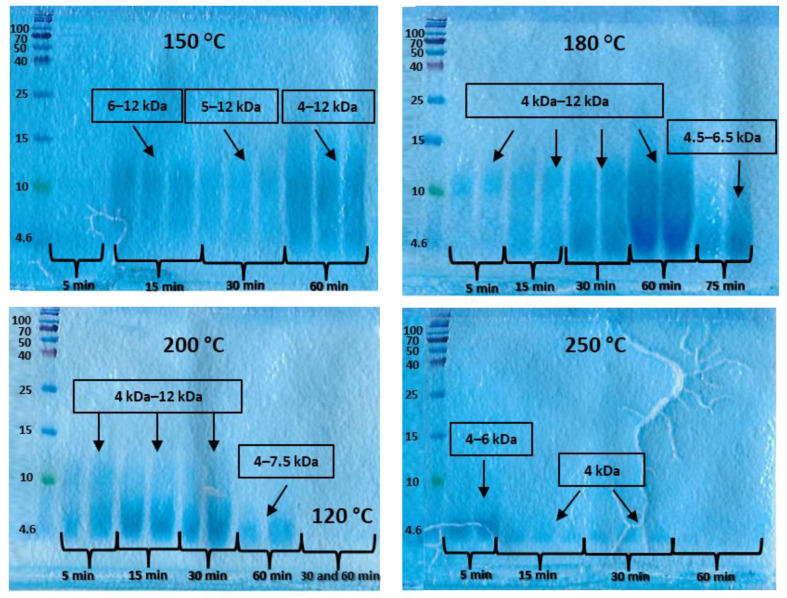
SDS-PAGE gels of isolated keratin from poultry feathers.

Figure 3 shows the SDS-PAGE electropherograms of the feather proteins isolated by SubCW at different temperatures and reaction times. In general, the molecular weight of the extracted proteins was in the range of 4 kDa and 12 kDa. It decreased with increasing temperature above 180 °C as long-chain proteins were further degraded to smaller molecules. As shown in Figure 3, the strong band between 4.5 and 12 kDa appeared after the hydrolysis of feathers at 180 °C and a reaction time of 60 min. Under these conditions, the extraction yield was also high (83.4%). Therefore, it can be concluded that the optimal conditions for isolating high molecular weight keratin molecules from feathers using SubCW are 180 °C and 60 min reaction time. The coloration of the bands is less intense at higher temperatures, and at a temperature of 200 °C and a reaction time of more than 30 min, the molecular weight begins to decrease. At a temperature of 250 °C and a reaction time of up to 30 min, the molecular weights are much lower and are only between 4 and 5 kDa. In contrast, at a reaction time of 60 min, no band is observed in the gels studied, which is a consequence of the degradation of the proteins to low molecular weight oligopeptides.

### 3.5. Content of Free Amino Acids in the Hydrolysate

**Figure 4:**
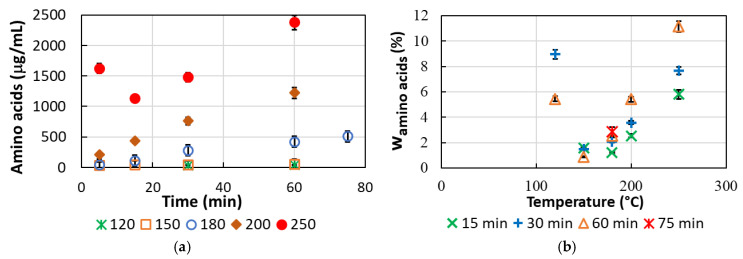
Content of amino acids in (**a**) aqueous solution, (**b**) dried proteinaceous product.

The major amino acids in poultry feathers are serine, glycine, leucine, proline, and alanine [8], and these are also the most abundant amino acids in the hydrolysate. The content of amino acids in the post-reaction solution was low at temperatures of 120 and 150 °C, ranging from 34.5–54.2 μg/mL (Figure 4). At temperatures above 150 °C, the amount of amino acids in the solution began to increase with increasing time and temperature, reaching a maximum value of 2378.9 μg/mL at 250 °C and 60 min, corresponding to 11.1 wt.% amino acids in the dried product.

**Figure 5:**
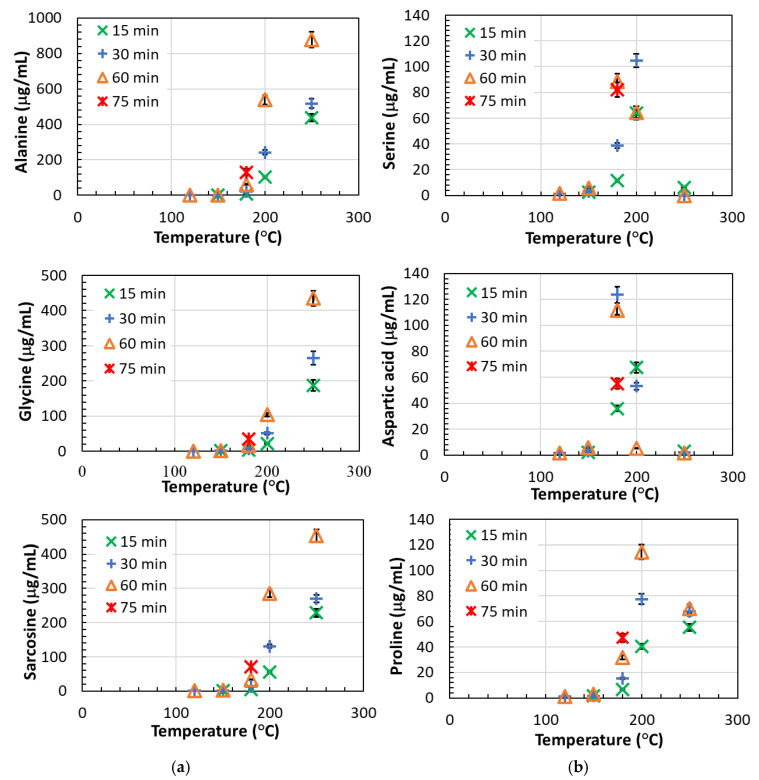
The influence of temperature at constant time on (**a**) concentration of alanine, glycine, and sarcosine and (**b**) on serine, proline, and aspartic acid in solution.

The amino acids detected in the highest amount were nonpolar amino acids such as alanine, glycine, and sarcosine (N-methylglycine), followed by nonpolar valine. For these amino acids, the time up to 60 min and the temperature up to 180 °C did not have much influence on the concentration. The increase in concentration is observed at temperatures above 180 °C, where the concentration increases with increasing temperature and time (Figure 5a). The highest amount of these amino acids was observed at 250 °C and 60 min, where these amino acids accounted for 82.4 wt.% of all amino acids in the solution (a total of 11.1 wt.% of amino acids in the dried product). Similar behavior is also observed for the nonpolar amino acids methionine, leucine, isoleucine, and phenylalanine, and for polar amino acids such as histidine, tyrosine, glutamic acid, dipeptide glycyl-proline, and α-aminobutyric acid; however, the concentrations were much lower (<82 μg/mL). Ornithine, lysine, allo-isoleucine, and tryptophan were also present in very small amounts (<34.5 μg/mL), and the concentration of these amino acids increases at temperatures above 200 °C.

Other amino acids detected in higher amounts in the solution were nonpolar proline, polar serine, and aspartic acid. However, they showed different behavior. Namely, their concentration increased with increasing temperature at a constant time, reached a maximum at a certain temperature, and then began to decrease as the temperature continued to rise. For aspartic acid, the concentration maximum is observed at 180 °C and 30 min, for proline at 200 °C and 60 min, and for serine at 200 °C and 30 min. This behavior results from the degradation of amino acids at higher temperatures and longer reaction times. In the solution obtained at 180 °C and 30 min, these amino acids accounted for 63.4 wt.% of all amino acids in the solution (a total of 2.1 wt.% of amino acids in the dried product). Similar behavior was observed for asparagine and threonine, however, their concentrations in the solution were much lower (<10.6 μg/mL). At temperatures of 120 °C and 150 °C, thioproline and α-aminopimelic acid, a lysine derivative, were found in very low concentrations (less than 2.5 mg/mL each), while glutamine was not detected. In addition to the abundance of amino acids in the protein molecule and their solubility in the reaction medium, the recovery of amino acids also depends on other factors such as the accessibility of the monomer in the protein molecule, the thermal stability of the peptide bonds associated with a monomer, and the thermal stability of the monomer [20].

The results obtained in the present work agree with previous studies dealing with the hydrothermal reactions of amino acids in SubCW and supercritical water (SCW) [14,39,40]. In the work of Klingler et al. [39], the decomposition of alanine and glycine was studied at 250 °C to 450 °C and 24 MPa. They found that glycine decomposes faster than alanine, but the conversion at 250 °C is very low (a few %). Sato et al. [40] studied the hydrothermal decomposition of five amino acids (alanine, leucine, phenylalanine, serine, and aspartic acid) in a temperature range from 200 to 340 °C and at a pressure of 20 MPa. They observed two main reaction pathways of amino acids under these conditions: decarboxylation to form carbonic acid (carbon dioxide) and amines and deamination to form ammonia and organic acids. They found that the decomposition rates of hydrophilic amino acids such as serine and aspartic acid tended to be higher than those of hydrophobic ones such as leucine, phenylalanine, and alanine. In addition, they observed the interconversion of amino acids, i.e., simple amino acids such as alanine and glycine were formed from the oxyamino acid serine. In contrast, glycine was formed from the oxyamino acid threonine [40]. In the work of Di Domenico Ziero et al. [20], SubCW hydrolysis of poultry feathers in a semi-continuous flow reactor was studied. They obtained the maximum concentrations of aspartic acid and serine at the highest flow rate (10 mL min^−1^) and the lowest temperature (210 °C), whereas the highest concentrations of glycine, valine, isoleucine, tryptophan, and methionine were obtained at the opposite extreme (250 °C and lowest flow rate of 5 mL min^−1^). These results are in agreement with the results of the present work. Similar results were obtained by Cheng et al. [14], who studied the hydrolysis of various protein-rich wastes in SubCW and found that for most amino acids the highest yields were obtained at temperatures between 200 and 290 °C.

### 3.6. Particle Size and Zeta Potential of Hydrolysate

**Figure 6:**
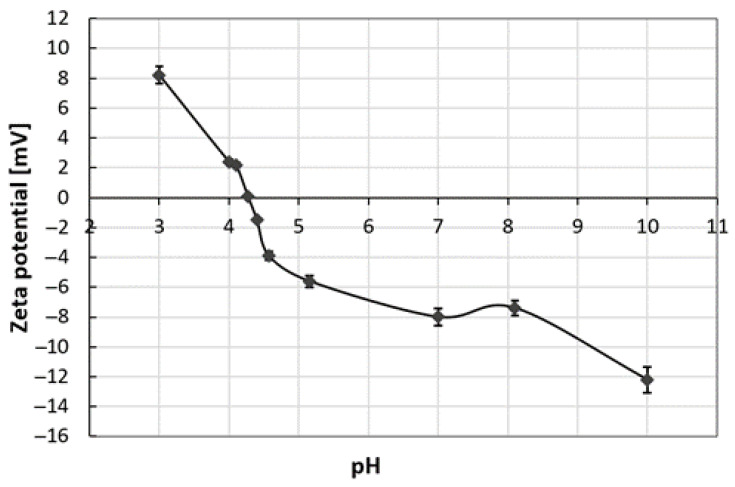
Zeta potential of hydrolysate as a function of pH.

Hydrothermal extraction of keratin from poultry feathers results in a macromolecular keratin solution in which smaller molecules such as amino acids and minerals are also present. The measured turbidity of the solution was 855 NTU, indicating that the solution is colloidal and contains particles on which light refracts. Keratin is a colloid-forming macromolecule. Its agglomeration with separate amino acids and other molecules (such as minerals) present in the hydrolysate can lead to the formation of multimolecular colloids [41]. To more accurately determine the particle size and charge of the prepared hydrolysate, zeta potential and particle size analyses were performed using laser diffraction. Figure 6 shows the zeta potential vs. pH curve.

Figure 6 shows the amphoteric character of the hydrolysate with the zero charges (isoelectric point) at pH = 4.4. Below this value, the solution exhibits a cationic character and, consequently, a positive zeta potential, which can be attributed to the presence of protonated amine groups of keratins and positively charged amino acids in the hydrolysate. Above the isoelectric point, the solution has a negative character, which can be attributed to the deprotonated carboxyl groups, which thus have an anionic character manifested in a negative zeta potential. This behavior is characteristic of proteins such as keratin, which have both amine and carboxyl groups disproportionate in their structure in the polymer chain [42]. It has to be noted that the absolute values of the zeta potential of the solutions are below ±30 mV regardless of the pH. This indicates the instability of the colloidal dispersion and the tendency of the particles to agglomerate. The keratin suspension is a very complex colloidal system in which, according to the DLVO (named after Boris Derjaguin and Lev Landau, and Evert Verwey and Theodoor Overbeek) theory, the stability is based on the competition of repulsive and attractive forces between particles [43]. According to our observations of the colloidal solution and the measured zeta potentials it seems that in our system, the attractive forces prevail over the repulsive forces, which leads to the agglomeration of the particles in the hydrolysate.

The hydrodynamic diameter (Table 1) of the particles in hydrolysate shows that the particles are largest at pH 7 and the hydrodynamic diameter is 7644 nm on average. The smallest hydrodynamic diameter of the solution particles is determined at pH 10, namely 2227 nm. At pH 3, the average hydrodynamic diameter of the particles is 5164 nm. The sizes are in the micrometer range due to the particles’ agglomeration and the presence of a generous group of macromolecules and molecules. Further dialysis would likely isolate the macromolecules from the smaller molecules, reducing agglomeration and increasing the stability of the purer keratin macromolecular solution.

The smallest particle size was obtained at pH = 3, where the zeta potential is about 8 mV, which allows a small amount of cationic charges (protonation of the primary amino groups) to prevent extensive agglomeration. At the isoelectric point, a higher particle size is achieved because there is no charge in the solution and the particles agglomerate strongly due to physically attractive interactions. At pH 7, the ZP of −8 mV is obviously not high enough to protect at least minimal particles from agglomeration. At pH 10, the negative ZP is −12 mV (anionic charge due to deprotonated carboxylic acids), which obviously has a more positive effect on the agglomeration process and hinders it.

### 3.7. Biocompatibility of Hydrolysate

**Figure 7:**
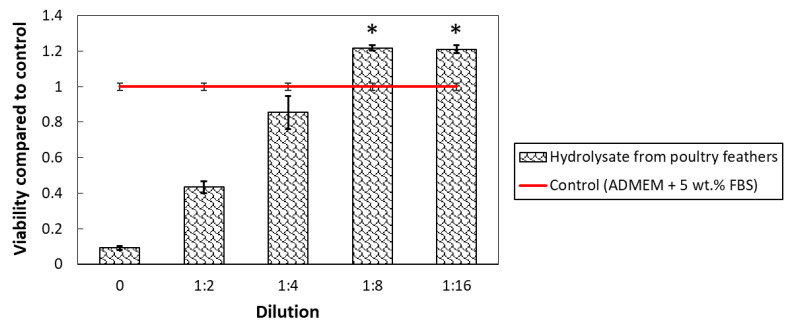
Cell viability of skin fibroblasts exposed to various keratin hydrolysate dilutions compared to the control sample (cell culture media). Asterisks indicate where differences between the sample and control were statistically significant (* *p* < 0.05).

Considering the source material for keratin extraction (poultry feathers, potentially contaminated, or containing potentially toxic waste materials) on the one hand, and the desire to use the extracted keratin in biomedical applications on the other, cell testing was performed. Being a new and potentially unsafe material for which no safe concentration range was known, a cytotoxicity test was performed using a series of dilutions of the initial sample with a concentration of 50 mg/mL (prepared in the cell culture media). The testing was performed according to ISO10993-5:2009 using skin fibroblasts. The cytotoxicity of the samples was determined quantitatively using the MTT assay and the results are shown in Figure 7.

The chosen starting concentration (50 mg/mL) was found to be too high for the exposed cells, which is seen from the low viability of the cells exposed to this sample. Considering ISO 10993-5:2009, which describes materials as toxic as soon as they lead to more than 30% cell death, the dilution of 1:2 (concentration of 25 mg/mL) was also toxic. However, already at the dilution of 1:4 (concentration of 12.5 mg/mL), the viability rose to over 80% compared to the control, indicating that such concentrations are already non-toxic. As expected, keratin can positively influence skin cell growth [44], which was also found for our hydrolysate in concentrations below 6.25 mg/mL (dilution of 1:8 and higher). In this concentration, the hydrolysate improved the skin fibroblast viability by over 20%, indicating not only its safety, but also a high potential to be used as part of novel formulations for treating skin-related diseases (either as drug delivery systems or materials for wound dressings).

**Figure 8:**
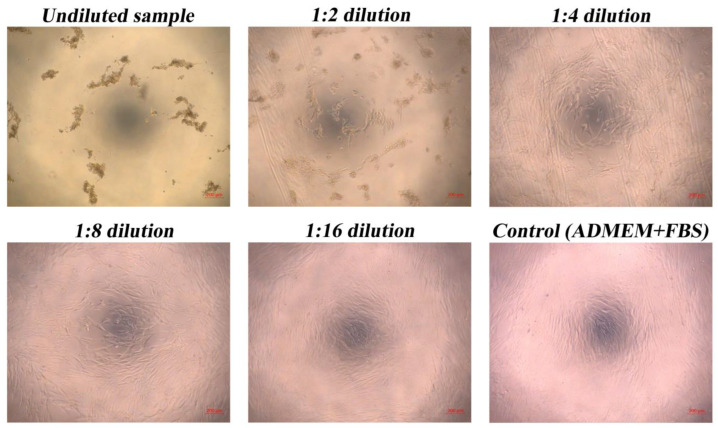
Optical micrographs of skin fibroblasts exposed to the different keratin hydrolysate dilutions and the control sample.

Finally, optical microscopy on the cells exposed to the undiluted hydrolysate and the differently diluted samples was performed. The micrographs (Figure 8) confirm the results from the MTT assay. Namely, at the 1:4 dilution (a concentration of 12.5 mg/mL of hydrolysate), the cells seem morphologically similar to the cells in the control sample (although they exhibit a lower confluence). From 1:8 dilution onwards, the optical micrographs of the cells exposed to the samples cannot be anymore distinguished from the control sample.

## 4. Conclusions

The results of this study show that subcritical water extraction represents an alternative green method for the isolation of high molecular weight keratin from poultry feathers. At the material-to-water ratio 1/20 g/mL, the optimal operating parameters for obtaining high molecular weight protein hydrolysate were determined to be 180 °C and 60 min. At these conditions, the yield of extraction was 83.4% (keratin extraction efficiency 91.7%), the molecular weight of the protein hydrolysate ranged from 4.5 to 12 kDa, and the content of amino acids in the dried product was a mere 2.5 wt.%. The dried hydrolysate obtained under optimal conditions showed no major differences in the chemical and secondary structure of the protein. The zeta potential measurement and particle size determination clearly show that the hydrolysate is a colloidal solution with a tendency for particle agglomeration, making it an interesting base material for preparing formulations for biomedical applications. Its promise for use in topical formulations was indicated using testing on human skin-derived fibroblasts with an increased viability of more than 20% for the extracted keratin concentrations of 6.25 mg/mL.

The results form an important basis for further development. In the future, it would be necessary to investigate the possibility of further optimization of the subcritical water extraction process by studying the influence of the material particle size and the material–water ratio, as well as the effects of further processing of the hydrolysate, e.g., by membrane separation processes, on the properties of the product. In addition, a more detailed analysis of the properties and processing characteristics of the product obtained by the subcritical water extraction method should be conducted and compared with the characteristics of products obtained by other methods to truly evaluate the potential of subcritical water technology.

## Figures and Tables

**Table 1:** Particle size of isolated keratin solution.

pH	Particle Size-Average Hydrodynamic Diameter [nm]
3	5164
4.4	6720
7	7644
10	2227

## Data Availability

Not applicable.
